# Current Perspectives on HIV-1 Antiretroviral Drug Resistance

**DOI:** 10.3390/v6104095

**Published:** 2014-10-24

**Authors:** Pinar Iyidogan, Karen S. Anderson

**Affiliations:** Department of Pharmacology, School of Medicine, Yale University, New Haven, CT 06520, USA; E-Mail: pinar.iyidogan@yale.edu

**Keywords:** HIV-1, antiviral therapy, drug resistance, evolution, lethal mutagenesis, resistance monitoring

## Abstract

Current advancements in antiretroviral therapy (ART) have turned HIV-1 infection into a chronic and manageable disease. However, treatment is only effective until HIV-1 develops resistance against the administered drugs. The most recent antiretroviral drugs have become superior at delaying the evolution of acquired drug resistance. In this review, the viral fitness and its correlation to HIV-1 mutation rates and drug resistance are discussed while emphasizing the concept of lethal mutagenesis as an alternative therapy. The development of resistance to the different classes of approved drugs and the importance of monitoring antiretroviral drug resistance are also summarized briefly.

## 1. Introduction

HIV-1 belongs to the retrovirus family and the etiologic agent of the acquired immunodeficiency syndrome (AIDS) that targets the human immune system [1]. In 2012, an estimated 35.3 million people lived with HIV while 1.6 million AIDS-related deaths occurred worldwide [2]. Currently, there is no cure for HIV infection. However, more than two decades of extensive scientific research has led to discovery of 26 FDA approved antiviral drugs with different mechanisms of action [3,4]. Nowadays, three or four drugs are combined as a multi-drug regimen, also known as highly active antiretroviral therapy (HAART). Most of the combination therapies include two nucleoside reverse transcriptase inhibitors (NRTIs) and one non-nucleoside reverse transcriptase inhibitor (NNRTI) or protease inhibitor (PI). Also, combinations of integrase or entry inhibitors with RT inhibitors and PIs are used as an alternative treatment strategy. Even though the current anti-HIV drugs and treatment strategies keep the viral load suppressed and the patients relatively healthy, the development of HIV drug resistance reduces or even eliminates the efficacy of antiretroviral treatment. HIV-1 possesses a high mutation rate and a high frequency of recombination, which could result in rapid emergence of drug-resistant variants when the viral replication is not sufficiently inhibited [5,6]. In this review, we will mainly focus on several topics to cover different aspects of HIV drug resistance: (i) the role of viral fitness in the treatment of HIV infection and evolution of resistance; (ii) lethal mutagenesis. Finally, we will also briefly discuss (iii) the currently available treatment options and (iv) the acquired drug resistance mutations during antiretroviral therapy as well as (v) the importance of clinical monitoring for drug resistance.

## 2. Viral Fitness and Its Influence on Drug Resistance

HIV-1 is a member of the retrovirus family having a high rate of mutation. Estimates of HIV mutation rate depend upon a number of factors including size of viral population and viral fitness of mutant strains. Earlier studies to measure the HIV mutation rate *in vivo* have determined a forward mutation rate of 3 × 10^−5^ mutations per target base pair per replication cycle [7]. A recent study highlights important caveats in experimental design for determining and comparing estimates of viral mutation rates [8]. Studies of HIV from clinical patient isolates suggest rapid viral turnover (10^8^ to 10^9^ virions per day) [9,10,11], large numbers of infected cells (10^7^–10^8^) [12], and a high level of recombination [13] The inherent high mutation rate by HIV generates a genetically diverse set of viruses usually from a single infecting viral genome [14]. The combinations of these genetically distinct HIV-1 subtypes exhibit different pathophysiological properties [15,16]. The viral “swarms” of heterogeneous populations are called quasispecies [17,18]. The quasispecies theory has linked the evolution trajectory of RNA viruses and viral pathology [19,20]. More insight into the theory revealed that the enhancement of viral mutagenesis above the error threshold could lead to error catastrophe [21,22,23] or extinction [24]. The high mutation rate of HIV-1 is crucial for adaptation to environmental changes like intracellular nucleotide concentrations, presence of mutagens, *etc*., that might affect the overall fidelity of the replication process [25,26]. More importantly, fidelity is vital for host cell infection efficiency and emergence of drug resistance [11,27]. *In vitro* measurements of HIV-1 reverse transcriptase (RT) fidelity indicate that de novo mutations are generated in the course of error-prone DNA synthesis while generating base substitutions, frame shifts, genetic rearrangements and hypermutations [28,29]. The main source for the high mutation rate of HIV-1 is due to the absence of 3'→5' exonucleolytic proofreading activity of HIV-1 RT [30,31]. Several kinetic studies of recombinant RT indicated a high level of misincorporation during polymerization, suggesting the contribution of RT for the hypermutability of HIV-1 [32,33]. Additionally, the fidelity of HIV-1 RT during the two polymerization steps, the minus-strand DNA synthesis from an RNA-template and plus-strand DNA synthesis from a DNA-template, plays a major role in determining the rate at which mutations occur. Another source for the additional mutations in HIV-1 genome could derive from the host RNA polymerase II during the synthesis of the plus-strand viral RNA [34,35]. However, O’Neil *et al.*, suggested that the majority of the mutagenesis in the viral genome is a result of misincorporation by HIV-1 RT rather than the host cell RNA polymerase II [36].

An increased mutation rate ensures the adaptability of virus in a changing environment such as utilization of ART, but the fine tuning of hypermutability before losing the viral fitness is crucial for HIV. One mechanism to tune HIV-1’s mutation rate is established through the interplay between the viral protein Vif (viral infectivity factor) and the host protein APOBEC3G, a cytosine deaminase [37,38,39]. The inhibitory effect of APOBEC3G in HIV-1 infection was first discovered in PBMCs that were infected with an HIV-1 strain lacking the Vif gene [40]. APOBEC3G has been shown to generate G-to-A hypermutations via deamination of cytosines to uracils during reverse transcription in newly synthesized minus-strand viral cDNA [41,42,43,44]. The presence of uracils in cDNA may then activate a cellular uracil-DNA glycolase causing the failure of reverse transcription. As a consequence, the proviral integration into the host genome is impaired in Vif-defective virus [42,45]. Additionally, the massive C-to-U conversion in the minus strand leads to ubiquitious G-to-A hypermutation of the proviral plus-strand cDNA after the integration of the proviral double stranded cDNA [46,47,48]. Thus, APOBEC3G is a critical element of an innate defense mechanism against HIV-1 infection. In essence, the host biologically induces lethal mutagenesis to defend itself against viral infections. HIV tackles the APOBEC3G-mediated hypermutagenesis with a counterattack. This involves binding of viral Vif protein to APOBEC3G and inducing polyubiquitination and degradation of APOBEC3G [49]. Recent advances in understanding this binding interaction between Vif and APOBEC3G [50] and the related mechanism of action studies will be important to develop novel antiviral drugs that targets the host and viral protein binding partners [51,52]. Moreover, a recent review presented how the mutation rate of HIV-1 could be modulated via the APOBEC3G-Vif interaction in order to adjust to the severe changes in environment and the evidence for the coevolution of both proteins through mathematical evolutionary modeling studies [53].

Besides the host-encoded enzymes altering the viral coding sequence and the error-prone retroviral replication process, genetic recombination is another process in the viral evolution of HIV-1 that contributes to viral fitness and avoidance of deleterious mutations while conserving the genomic information under the selective pressure such as antiviral drug treatment [54,55]. Mulder *et al.*, showed that only the recombination of hypermutated HIV-1 with wild type (WT) virus generated replication-competent drug resistant viruses [56]. The recombination takes place primarily during minus-strand or rarely during plus-strand DNA synthesis and associated with a single crossover [57,58,59]. Earlier studies showed that RT switches templates between 2 to 13 times per genome replication and an average of three crossovers is detected per genome in the course of one cycle [55,59,60]. Additionally, the number of crossovers increases to an average of 9 and 30 per replication cycle for T lymphocytes and macrophages, respectively [61]. The recombination ability provides an efficient mechanism to redistribute mutations while elevating variation in the viral population. Generating multi-drug resistant variants in a viral population through the frequent recombination cycles hinders antiviral treatment [62,63,64,65]. As noted above, this characteristic escape route that HIV-1 exploits should be further investigated in the context of hypermutation. At this point, lethal mutagenesis could be an alternative strategy to fight against the multi-drug resistance problem through artificially increasing mutations with the use of chemical mutagens to a point that even the recombination-mediated repair of defective retroviral genomes would no longer function and eventually lead to error catastrophe.

## 3. Lethal Mutagenesis

The inherent infidelity of HIV-1 RT generates a wide diversity of mutated progeny and this property is crucial for viral evasion from the host immune defense or ART, and provides an extraordinary adaptability for the virus [66]. However, a high error rate of HIV-1 RT also leads to deleterious effects on the viral fitness after reaching a certain threshold. For instance, exploring the effects of mutations in the protein-coding regions of HIV-1 genome by sequencing revealed that all of the mutations were nonsynonymous and almost half of these were deleterious due to some premature stop codons [67]. Therefore, it has been proposed that the use of a mutagenic nucleotide analogue should slowly accumulate mutations over multiple rounds of replication, which then leads to elevated mutational load until the induction of lethal mutagenesis via generation of defective or non-infectious viral particles.

**Figure 1:**
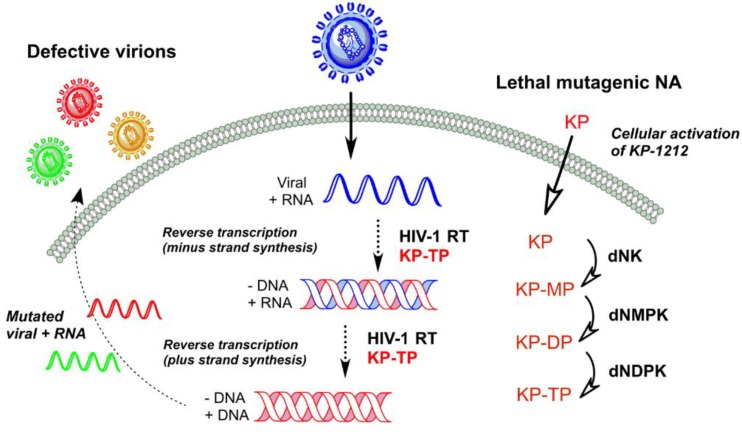
Schematic representation of HIV-1 lethal mutagenesis.

The concept of lethal mutagenesis introduced by Loeb *et al.*, was first introduced to HIV-1 by using a mutagenic deoxynucleoside analogue, 5-hydroxy-2'-deoxycytidine (5-OH-dC) that reduces the fidelity of replication [68]. When the error threshold is approached with a mutagenic agent, small increases in mutation rates give rise to large declines in viability [69]. However, these mutagenic nucleosides need to be metabolically activated to their corresponding 5'-triphosphate forms by cellular kinases in order to exhibit their mutagenic activities after the penetration of these nucleoside analogues (NA) into the infected cells via passive diffusion or the nucleoside transporters as highlighted in Figure 1 [70]. Interestingly, the initial phosphorylation step is considered to be the rate-limiting step compared to the subsequent phosphorylation steps catalyzed by deoxynucleoside mono- (dNMPK) and diphosphate kinases (dNDPK) to afford the active nucleoside analogue triphosphates (NA-TP) responsible for the mutagenic activity [71]. Subsequently, the pharmacologically active mutagenic nucleoside analogue triphosphate is incorporated into the nascent viral DNA strand by HIV-1 RT during minus-strand DNA synthesis, which would generate mispairs in the subsequent plus-strand DNA synthesis as shown in Figure 1. Consequently, incorporated mutagenic nucleosides lead to increased mutation rates. Similarly, the mutagenic ribonucleoside analogues are incorporated into viral RNA transcripts upon phosphorylation by cellular RNA polymerase II, resulting in base mispairing during subsequent RT-catalyzed minus-strand DNA synthesis in newly infected cells [72]. The removal of mismatched nucleotides is unlikely due to inefficient excision activity by the DNA repair enzymes from the RNA-DNA hybrid duplexes [73]. Therefore, mutations accumulate in the newly synthesized viral RNA genome per replication cycle and cause an irreversible genetic meltdown of the virus population until it reaches a point such that no functional viral proteins can be produced. Fortunately for the virus, the vast population size, genome structure and tight regulation of adaptive requirements have placed the virus populations very close to the extinction threshold. Hence, it has been suggested that altering the error frequency through a lethal mutagen could be ineffective for fully extinguishing the viral population or diminishing the spread of virus [24].

The early HIV-1 studies with 1 mM 5-OH-dC resulted in irreversible loss of viral infectivity and by serial viral passage at round 17 no detectable levels of p24 were present [68]. On the contrary, no loss in viral titer was detected in the absence of the mutagenic analogue. 5-OH-dC shows its activity through presenting imino and amino tautomers [74] at physiological conditions and then base pairing with guanosine and adenosine. The sequence analyses of PCR products from the passage 16 virus indicated a 6-fold increase in the frequency of G-to-A transitions. The preferred adenosine over guanosine pairing of 5-OH-dC is a result of the imino tautomer frequency, which regulates the switched base pairing. The additional serial passage experiments with 5-OH-dC showed similar declines in viral titer and confirmed the G-to-A transitions [75]. Moreover, 5-OH-dC was non-toxic to the host genome since its incorporation was as low as one molecule per 1 × 10^5^ nucleotides in cellular DNA and also no mitochondrial toxicity was detected [76]. The potential for utilizing ribonucleoside derivatives as mutagenic nucleoside analogues has been explored for 5-Azacytidine (5-AzaC) to induce HIV-1 lethal mutagenesis. 5-AzaC is clinically used to treat myelodysplastic syndrome [77] and has been also shown to inhibit HIV-1 replication in human CEM cells [78]. 5-AzaC could induce lethal mutagenesis through increasing the G-to-C transversions at drug concentrations that has no cell toxicity [79,80]. According to the proposed mechanism for 5-AzaC, ribonucleotide reductase converts 5-AzaC into its 2'-deoxyribonucleoside form that is subsequently phosphorylated and then the triphosphate form gets incorporated into viral DNA during reverse transcription by RT. Once incorporated into DNA, a water molecule attacks to the C6 position of the mutagenic nucleobase and cause ring opening while resulting in G-to-C transversions during the plus strand synthesis [81,82]. No significant adverse effects to the host cell were detected by 5-AzaC, which could be due to the possible detection by the host DNA machinery and subsequent elimination from genomic DNA in case of incorporation [83].

**Figure 2:**
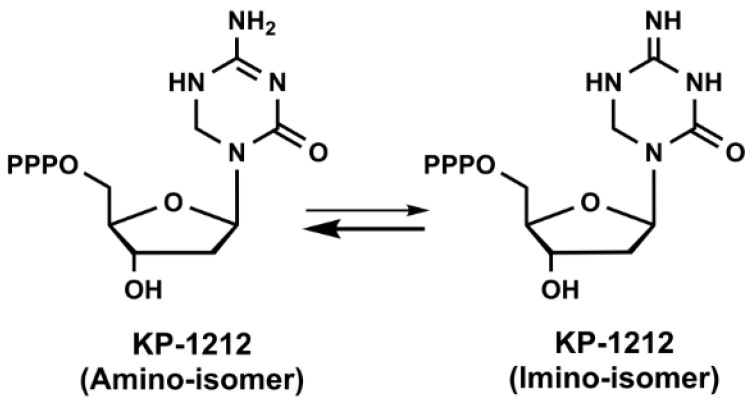
Chemical structures of KP-1212 in amino and imino isomer.

KP1461 (N4-heptyloxycarbonyl-5,6-dihydro-5-aza-2'-deoxycytidine) is a prodrug of KP1212 (5-aza-5,6-dihydro-2'-deoxycytidine, Figure 2), which is another mutagenic deoxycytidine analogue that induces acceleration of HIV error rate during viral replication by RT [84]. KP1212 possesses ambiguous base pairing ability that elevates the number of random mutations in the viral genome. This drug tautomerizes between the amino and imino forms in the nucleobase moiety as shown in Figure 2, which could result in base pairing with guanine and adenine. Koronis Pharmaceuticals (Redmond, WA, USA) carried KP1461 into three Phase I and one Phase II clinical trials for the treatment of HIV. KP1461 is converted to KP1212 by liver enzymes and sequentially phosphorylated into its triphosphate form. Kinetic analysis of KP1212-TP incorporation by HIV-1 RT showed a 19- and 12-fold reduction in incorporation efficiency compared to the natural substrate (dCTP) for DNA and RNA templates, respectively [85]. The initial serial passage experiments of KP1212 reported an increase in the mutation and viral annihilation by passage 13 at 10 μM [84]. The sequencing data from this study revealed a predominance of G-to-A and A-to-G transition mutations and to a lesser extent C-to-T and T-to-C transitions with no significant increase in transversions. These transitions are consistent with the coexistence of both tautomers as explained above. Most of the NAs cause mitochondrial toxicity during the long-term treatment [86]. Therefore, we have studied the potential mitochondrial toxicity of KP1212 by *in vitro* experiments [85]. Our results demonstrated that human mitochondrial DNA polymerase (Pol γ) could incorporate KP1212 into the DNA template as well as HIV-1 RT, which makes it potentially toxic to mitochondria. However, Pol γ could efficiently excise the incorporated KP1212-5'-monophosphate (KP1212-MP) via its proofreading exonuclease activity. Moreover, mitochondrial toxic effects were also tested by a lactate assay in human CEM cells along with a test of mitochondrial DNA (mt DNA) synthesis after KP1212 treatment [84]. According to the results, only 6% decrease in mt DNA synthesis was detected with 320 μM KP1212 and lactic acid quantities were insignificant up to 1 mM of drug. The calculated *K*_i_ values of KP1212-TP for HIV-1 RT and Pol γ were 95 μM and 28 μM, respectively and the discrimination ratio between dCTP and KP1212-TP was 27 for Pol γ and 78 for RT enzyme. Additionally, the incorporation efficiency of KP1212-MP with HIV-1 RT and Pol γ is 11-fold and 26-fold lower than dCMP, respectively.

KP1461 successfully completed Phase Ia and Phase Ib human clinical trials with no serious toxicity concerns for healthy and HIV-infected subjects [87]. In the Phase I b study, HIV positive subjects demonstrated a statistically significant drop in viral load. After the encouraging results of Phase I study, KP1461 entered an open label Phase IIa clinical trial including chronically infected and treatment-experienced patients [88]. In this Phase IIa trial, salvage HIV patients received 1600 mg of KP1461 as a monotherapy twice per day for 124 days. While these patients entered the trial with pre-existing drug resistance mutations, the analysis of clinical data exhibited no significant decrease in plasma viral load, and the number of resistance mutations was not increased by KP1461. Additionally, no new resistance mutations were selected during this short-term clinical study, yet the mutation spectrum was altered for HIV [89]. The sequenced viral genes originating from individual HIV-1 RNA templates revealed an excess of A-to-G and G-to-A transition mutations, and to a lesser extend T-to-C and C-to-T mutations compared to control group. It has been suggested that KP1461 could be coadministered with other inhibitors in addition to monotherapy, since the mutagen will further weaken the already poor viral fitness in failing ART regimens. In conclusion, KP1461 could offer a novel mechanism of action and invaluable advancement in the treatment of HIV-1 infection.

## 4. Antiretroviral Drug Classes and Acquired HIV-1 Drug Resistance

### 4.1. FDA Approved NRTIs and Mechanisms of Resistance

**Figure 3:**
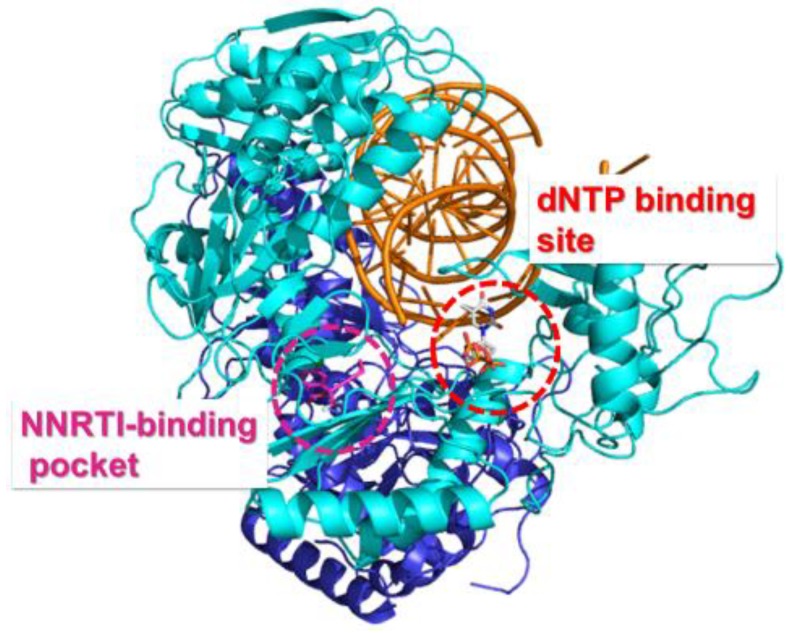
Overall view of the HIV-1 RT/DNA/dNTP ternary complex showing the corresponding binding sites of non-nucleoside reverse transcriptase inhibitor (NNRTI) (circled in magenta, efavirenz (EFV) is shown as magenta sticks) and NRTI (circled in red, dNTP is shown as sticks). The p66 and p51 subunits are represented in cyan and blue, respectively. PDB 1RTD [94].

NRTIs play a central role in HAART and have been utilized extensively in the fight against HIV-1 infection [90]. NRTIs are administered as inactive prodrugs and require metabolic conversion to their corresponding active 5'-triphosphate forms by host cellular kinases [91]. These pharmacologically active drugs are efficiently incorporated into the viral DNA at the 3'-end as an NRTI monophosphate by HIV-1 RT while competing with the natural deoxynucleoside triphosphates (dNTPs). NRTIs bind at the nucleotide binding site as illustrated in Figure 3.Upon incorporation, these NAs inhibit the elongation of viral DNA chain due to the lack of a 3'-hydroxyl group or an altered sugar moiety that prevents the incorporation of next incoming nucleotide. The commonly used pyrimidine analogues are 2',3'-dideoxy-2',3'-didehydrothymidine (d4T, stavudine) and 3'-azido-2',3'-dideoxythymidine (AZT, zidovudine) as thymidine (T) analogues, and 2'-*deoxycytidine* (dC) analogues are β-l-(−)-2',3'-dideoxy-3'-thiacytidine (3TC, Lamivudine), and β-l-(−)-2',3'-dideoxy-5-fluoro-3'-thiacytidine (FTC, emtricitabine). On the other hand, purine analogues are β-d-(+)-2',3'-dideoxyinosine (ddI, didanosine) as 2'-*deoxyadenosine* (dA) analogue, (−)-(1S,4R)-4-[2-amino-6-(cyclopropylamino)-9H-purin-9-yl]-2-cyclopentene-1-methanol (ABC, abacavir) as 2'-*deoxyguanosine* (dG) analogue, and *R*-9-(2-phosphonomethoxypropyl) adenine (TDF, tenofovir) as a chemically unique acyclic phosphonate nucleotide analogue. There are two main mechanisms underlying HIV-1 drug resistance towards NRTIs [91]. The first mechanism involves discriminatory mutations that would weaken the binding affinity of the NRTIs while retaining the binding efficiency of the corresponding natural dNTPs. As a result, the incorporation of NRTIs into the viral DNA diminishes and the virus continues to proliferate. The other common resistance mechanism is nucleotide excision representing the reversal of the polymerization reaction in order to restore the DNA synthesis. In this mechanism, RT utilizes ATP or inorganic pyrophosphate (PP_i_) as a co-substrate to remove the incorporated nucleoside analogue monophosphate (NA-MP) that terminates the DNA elongation [92,93].

#### 4.1.1. NRTI Specific Discriminatory Mutations

**Figure 4:**
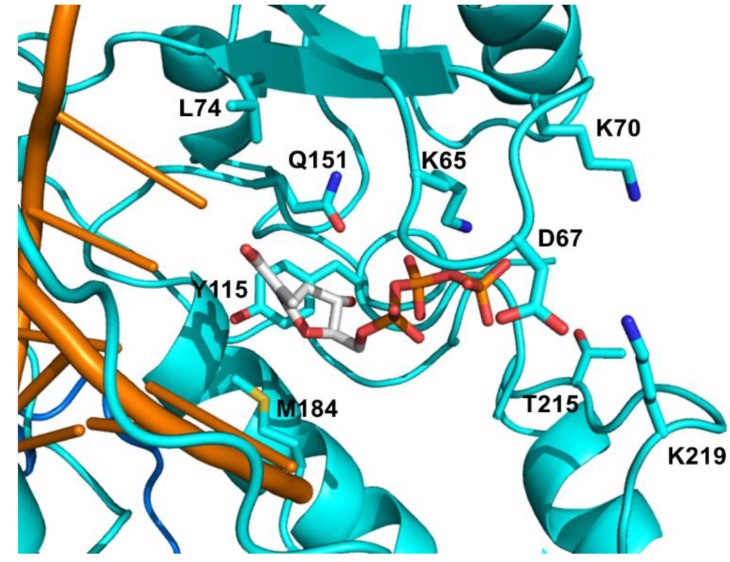
Detailed view of the NRTI binding pocket. The drug resistant mutation sites are shown in cyan sticks. dNTP is highlighted in sticks. PDB 1RTD [94].

The major NRTI mutations against the FDA approved drugs are listed in Table 1 and highlighted in Figure 4. The discrimination pathway is represented by K65R, L74V, Q151M, and M184V/I mutations, which diminish affinity of RT for specific NRTIs with no significant change in corresponding natural dNTP affinity. The amino acid K65 interacts with the γ-phosphate of the incoming dNTP [95] and K65R mutation mediates discrimination through reduced polymerization rate (*k*_pol_) for the incorporation of all NRTIs [96,97,98]. Viruses with the K65R mutation showed decreased susceptibility for TFV, 3TC, FTC, ddI, and ABC between 2- to 7.7-fold, but no significant resistance for AZT and d4T [99,100].

The side chain of M184 interacts with the ribose moiety and the nucleobase of the 3'-nucleotide in the primer, however substituting methionine with β-branched amino acids (valine, isoleucine, threonine) makes an interaction with the dNTP sugar moiety [95]. According to this structural information, binding of NRTIs with β- or L- ring configurations like 3TC and FTC would be sterically hindered by M184V/I mutations [101,102]. These mutations are selected during 3TC and FTC treatment and confer a high level of resistance to them via interfering with binding of 3TC-TP and FTC-TP [103]. Additionally, M184V/I mutations decrease the viral replication capacity in the presence of low dNTP concentrations [104,105]. The clinical data has indicated that the prevalence of the M184V/I resistance mutation was significantly lower in patients who received FTC and TDF compared to those who received 3TC and TDF [106].

**Table 1:** Major NRTI Resistance Mutations.

Consensus	Amino acid mutations associated with						
	FTC	3TC	TDF	ABC	ddI	d4T	AZT
M41						L	L
K65	R	R	**R**	**R**	**R**	R	L
D67						N	N
K70	E	E	E	E	E	R	R
L74	**V**,**I**	**V**,**I**		V,I	V,I		
Y115			F	**F**			
M184	**V**,**I**	**V**,**I**		V,I	V,I		
L210						W	W
T215						Y, F	Y, F
K219						Q,E	Q,E

The resistance mutations are compiled from the updated International Antiviral Society-USA (IAS-USA) and Stanford HIV drug databases. Mutations that are highlighted in bold represent the reduced susceptibility or virological response.

Q151 is located in the palm subdomain of the RT, where it contacts the 3'-OH of ribose moiety and the nitrogen base of the incoming dNTP [95]. The interaction with the 3'-OH group allows the Q151M mutant RT to better discriminate between natural dNTPs possessing 3'-OH, and NRTIs lacking this hydroxyl group [99]. Consequently, the Q151M mutation results in nucleotide discrimination through a reduction in the *k*_pol_ values of NRTI incorporation [97,107,108]. Q151M and accompanying other mutations including A62V, V75I, F77L, and F116Y confer multi-NRTI resistance and this multiple mutational pattern is called Q151M complex. The Q151M complex viruses show high-level resistance to all NRTIs (>11-fold) except 3TC, FTC and TDF with minimal susceptibility changes around 2-fold [99,100,109]. Several other mutations around the β3–β4 hairpin loop, position 62-to-72, have been associated with the Q151M complex. These mutations include T69N, K70G/Q or the deletion of T69 residue [110,111]. Residue K70 is located in the β3–β4 hairpin loop and K70E confers resistance to TDF by lowering its incorporation rate while preserving the binding affinity [98]. L74 interacts with the template strand at position +1 [112]. L74V mutation is selected by ddI and abacavir by reducing their *K*_i_ values [99,113]. In the presence of M184V, L74V confers reduced susceptibility to ABC and ddI [114] and appears to be associated with an inferior virologic response to TDF-based regimens [115].

#### 4.1.2. Excision of NRTIs

Another common resistance mechanism observed in HIV-1 RT involves the selective removal of NRTIs from the 3' end of a chain-terminated viral DNA primer [116]. The removal of the chain terminator leaves the primer terminus compatible for further nucleotide incorporation again. In this excision mechanism, ATP and PP_i_ nucleophilically attack the terminal phosphodiester bond of the chain-terminated primer, yielding a dinucleoside tetraphosphate [117] or nucleoside analogue triphosphate (NA-TP) [118], respectively. NRTI excision represents the reversal of the polymerization reaction in order to restore the DNA synthesis and it has been suggested that ATP is the physiologically relevant pyrophosphate donor for this reaction. Since AZT is the most efficiently removed NRTI among all NRTIs, excision of AZT has been extensively studied to explore the phosphorolytic removal mechanism [119]. Previous biochemical studies showed that a set of mutations including M41L, D67N, K70R, L210W, T215F/Y, and K219E/Q lead to high-level resistance to AZT and combinations of these mutations are designated as thymidine analogue resistance mutations (TAMs) or AZT resistance mutations (AZTr) [120,121]. Additionally, these mutations confer cross-resistance to AZT and d4T, as well as to most NRTIs to varying degrees [122]. Boyer *et al.*, suggested that some of the TAM mutations increase the AZT removal via enhancing the binding of ATP [123]. The excision of AZT-5'-monophosphate (AZT-MP) from terminated primer by ATP results in the formation of AZT-5'-tetraphospho-5'-adenosine (AZTp4A) as an excision product [124]. The excision reaction could be blocked by the next complementary dNTP, which forms a dead-end-complex (DEC) [125,126]. Once the dNTP occupies the nucleotide binding site (N-site) of the RT ternary complex, the chain terminator located at the end of a primer moves to the priming site (P-site), thus preventing the phosphorolytic excision of NRTIs and blocking the viral DNA synthesis [127]. TDF and d4T are the other commonly studied NRTIs for phosphorolytic excision reactions. While AZT-MP excision is highly dependent on TAMs, TDF removal is predominantly sensitive to DEC formation [128]. Moreover, we previously showed that ATP-mediated excision of TDF is more efficient using the polypurine tract (PPT) viral sequence than the primer binding site (PBS) sequence [129]. Introducing an NNRTI like EFV also forms a ternary complex resembling DEC and blocks the removal of TDF from the terminated primer. A recent crystal structure of AZTr mutant RT (M41L, D67N, K70R, T215Y, and K219Q) bound to both the AZTp4A and primer/template revealed that T215Y and K70R make specific binding interactions with AZTp4A and combinations of AZTr mutations form a high-affinity ATP binding site on the enzyme [130]. As a result, these mutations coordinate the excision product better than WT RT since none of these interactions are present in the WT structure [95]. Earlier studies demonstrated that dissociation of AZTp4A is the rate limiting step for continuing DNA synthesis [131] and the dinucleoside tetraphosphates like AZTp4A serve as substrates for RT and chain terminate the DNA elongation [132].

#### 4.1.3. Multi-NRTI Resistance Mutations

Once RT acquires the TAM complex mutations with high resistance to thymidine analogues, it could also develop further mutations like T69 insertion complex and become resistance to variety of NRTIs [133]. HIV-1 RT variants with the insertion of dipeptides (Ser-Ser, Ser-Gly or Ser-Ala) near the residue T69 in the finger subdomain and several other mutations like M41L, A62V, T69S, K70R and T215Y decrease the viral susceptibility to AZT and d4T while enhancing the ATP-mediated excision activity of RT for both NRTIs [134]. Biochemical studies showed that the insertion mutations mostly destabilize the DEC formation with the terminated primer/templates including various NRTIs as chain terminators in the presence of physiological dNTP concentrations [135,136]. The other two main multi-NRTI resistance mutation groups including Q151 and TAM complexes are explained in the previous subsection. Patients treated with thymidine analogues develop two TAM pathways with high resistance to all NRTIs: TAM 1 (T215Y linked; M41L, L210W, and T215Y) and TAM 2 (T215F linked: D67N, K70R, K219E/Q, and T215F) [122]. Table 2 summarizes the multi-NRTI resistance mutations.

**Table 2:** Multidrug Resistance Mutations.

Consensus	Amino acid mutations associated with		
	69 insertion complex	Q151 complex	TAMs
M41	L		L
A62	V	V	
D67			N
T69	Insert		
K70	R		R
V75		I	
F77		L	
F116		Y	
Q151		M	
L210	W		W
T215	Y,F		Y,F
K219	Q,E		Q,E

The resistance mutations are compiled from the updated International Antiviral Society-USA (IAS-USA) and Stanford HIV drug databases.

#### 4.1.4. Investigational NRTIs and Emergence of Potential Resistance Mutations

Two newly developed NRTIs showed improved safety and antiviral activity profiles with minimal drug resistance problems. The first drug is an adenosine analogue, 4'-ethynyl-2-fluoro-2'-deoxyadenosine (EFdA), currently in Phase I clinical trials with superior activity profile compared to the approved NRTIs [137]. EFdA’s antiviral activity is in the pM range against WT virus and more importantly, this new drug suppresses the replication of various multidrug-resistant HIV variants [138]. EFdA is unique in its structure and mechanism of action since EFdA-TP can be incorporated efficiently by its 3'-OH group and block the translocation of the primer strand in the RT complex [139]. EFdA is not only potent against several multidrug-resistant variants such as K65R, L74V, M41L/T215Y, and Q151M complex [137,140], but also show favorable toxicity profiles and stability in plasma [141]. We also determined that the mitochondrial toxicity of this new analogue is neglible since EFdA-TP is a poor substrate for human mitochondrial DNA polymerase γ [142]. Furthermore, our pre-steady-state kinetics studies showed that RT preferentially incorporates EFdA-TP over the natural dATP substrate and promoted delayed chain termination with unaffected fidelity [143]. *In vitro* resistance studies illustrated that novel combinations of mutations are selected by EFdA, in which the triple mutation, I142V/T165R/M184V, showed the highest resistance profile [137].

The next drug is a derivative of d4T, 2',3'-didehydro-3'-deoxy-4'-ethynylthymidine (Ed4T), in Phase IIb clinical trial. Unlike its predecessor d4T, Ed4T possess potent antiviral activity against viruses carrying K65R and Q151M resistance mutations and much less inhibitory effects on mitochondrial DNA synthesis [144,145]. More importantly, viruses with the entire Q151M complex mutations maintained susceptibility to Ed4T [146]. However, addition of M184V to this complex significantly decreased the susceptibility to this drug. Additionally, *in vitro* studies have demonstrated that M184V alone and P119S/T165A/M184V conferred 3- and 130-fold resistance to Ed4T, respectively [147]. Clinical isolates with T69 insertion complex including T210W and T215Y exhibited a high resistance to Ed4T [146].

### 4.2. NNRTI Resistance

**Figure 5:**
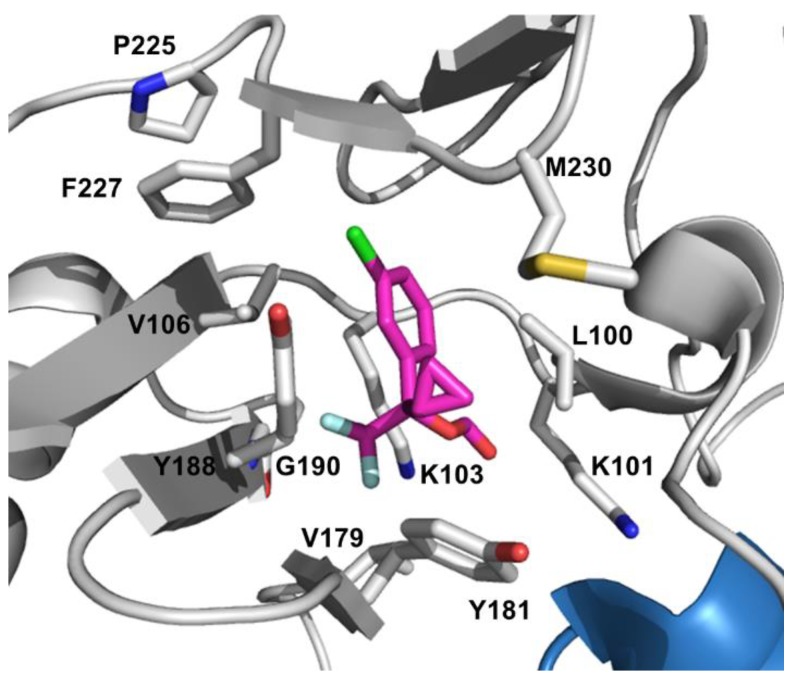
Detailed view of the NNRTI binding pocket. EFV is shown as magenta sticks and the EFV-resistant mutation sites are shown in white sticks. PDB 1FK9 [152].

Unlike NRTIs, NNRTIs possess diverse chemical composition and do not require intracellular activation to show their pharmacological activities [91]. Currently, there are five NNRTIs approved for treating HIV-1 infection: Nevirapine (NVP), Delavirdine (DLV), Efavirenz (EFV), Etravirine (TMC125, ETR), and Rilpivirine (TMC 278, RPV). NNRTIs bind to an allosteric hydrophobic site, approximately 10 Å away from the polymerase active site, which is unique to HIV-1 RT and absent in host cell polymerases [148]. NNRTIs are non-competitive inhibitors since they do not interfere with dNTP binding, but rather induce conformational changes in the vicinity of the active site upon binding to the hydrophobic NNRTI-binding pocket (NBP), thus blocking the required alignment of substrates for the phosphodiester bond formation (Figure 5) [149,150,151].

NNRTIs have become a crucial part of HAART because of their minimal side effects and less toxicity profiles compare to NRTIs [153]. However, emergence of resistance and cross-resistance could diminish or completely abolish their therapeutic efficacy. There are several factors play an important role in NNRTI resistance such as the ease of sequence variability in residues aligning the NBP since no conserved sequence is required compare to the dNTP-binding site, and the effect of resistance mutations on drug susceptibility and viral fitness [154]. Table 3 summarizes the most common clinically significant NNRTI-resistance mutations. First-generation inhibitors including NVP [155], and DLV [156], have a lower genetic barrier to resistance than the second-generation inhibitors such as EFV [157]. Third generation compounds ETR [158] and RPV [159] have enhanced potency and effectiveness on drug-resistant mutants. Resistance mutations can emerge relatively quickly against the first-generation NNRTIs. Primary resistance mutations that are selected by these drugs are all located in the NBP, and reduce the binding affinity of the inhibitors or block their access to NBP [160]. This hydrophobic pocket is formed by amino acids L100, K101, K103, V106, T107, V108, V179, Y181, Y188, G190, F227, W229, L234, and Y318 of p66 and E138 of p51 [161,162].

**Table 3:** Major NNRTI Resistance Mutations.

Consensus	Amino acid mutations			
	NVP	EFV	ETR	RPV
V90			I	
A98			G	
L100	I	**I**	**I**	**I**
K101	**P**,E,H	**P**,E,H	**P**,E,H	**P**,E,H
K103	**N,S**	**N,S**		
V106	**A,M**	**M**, A	I	
V108	I			
E138			A,G,K,Q	**K**,A,G,Q,R
V179	D,E,F	D,E,F	D,F,T,E	L,D,E,F
Y181	**C**,**I**,V	C	C,**I**,**V**	C,**I**,**V**
Y188	**C**,**L**,H	**L**,**C**,H		**L**
G190	**A**,**S**,**E**,**Q**	A,**S**,**E**,**Q**	S,A,E,Q	A,S,E,Q
H221				Y
P225		H		
F227	L,C	L,C	C	C
M230	**L**	**L**	L	I,L

The resistance mutations are compiled from the updated International Antiviral Society-USA (IAS-USA) and Stanford HIV drug databases. Mutations that are highlighted in bold represent the reduced susceptibility or virological response.

#### 4.2.1. Resistance to First-generation NNRTIs

DLV is precluded from clinical treatment due to its limited efficacy and inconvenient dosing requirements [163]. NVP is also no longer recommended as a part of first-line regimen NNRTI because of its toxicity profile and the availability of better options [164]. Currently, EFV is the only earlier-generation NNRTI that is used in combination therapies to treat HIV infection. The three most common single mutations that confer high-level resistance to all of the first-generation NNRTIs are K103N, Y181C and G190A and these mutations cause clinical failure [165].

The K103 position is located at the edge of the NBP and in the vicinity of the entrance to this pocket with its side chain pointing out. Structural data suggests that the K103N mutation forms a hydrogen bond to Y188 residue in the unliganded RT and this additional hydrogen bond keeps the entrance to the pocket closed, thus interfering with the ability of NNRTIs to enter the pocket [166,167]. These observations also explain the slow binding kinetic results of the inhibitors to K103N mutant enzyme [166]. The K103S mutation occurs less frequently in patients and causes resistance to DLV, NVP, and EFV [168]. Amino acid K101 also resides at the site of NNRTI entry part of the pocket and K101P mutation confers large reductions in susceptibility to all the currently approved NNRTIs [169].

NVP like inhibitors maintain a “butterfly-like” binding mode in the pocket and amino acids Y181 and Y188 stabilize the NVP binding through stacking interactions between their aromatic side chains and the pyridine groups on the inhibitor [170]. The crucial contribution of these aromatic ring stacking interactions to the NNRTI binding energy explains the drastic decrease in inhibitor binding once Y181C and Y188C mutations emerge [171]. Y181C causes 35-, 161-, and 3-fold reduced susceptibility to DLV, NVP, and EFV, respectively [172]. Even though the Y181C-mediated EFV resistance is at the low level, selection of the more resistant K103N mutation causes treatment failure with EFV [173]. This K103N/Y181C double mutant leads to cross-resistance to all NNRTIs in this group [174]. Y188L mutation exhibited cross-resistance to all the earlier-generation NNRTIs with high-level resistance to NVP and EFV [175].

The G190A mutation leads to high resistance to NVP and mid-level resistance to EFV through the steric hindrance between the alanine side chain and the bound NNRTI structure [172]. On the other hand G190A/S causes hyper susceptibility to DLV [176]. The less frequent G190E mutation confers high-level resistance to EFV and NVP while diminishing the polymerase activity simultaneously [177]. L100I is another steric hindrance mutation besides G190A and confers resistance to all NNRTIs [172].

#### 4.2.2. Resistance to Later-generation NNRTIs

ETR and RPV are diarylpyrimidine (DAPY) derivatives and rationally developed using structure-based design approach in order to circumvent the inhibitory effects of the common NNRTI-resistance mutations [178]. Contrary to the earlier-generation NNRTIs with a low genetic barrier, second-generation NNRTIs possess an increased genetic barrier to the development of resistance due to their conformational flexibility in the NBP [158,179]. These next generation inhibitors adopts a horseshoe shape in the pocket, and maintain their binding interactions through reorienting or repositioning at the pocket even in the presence of the emerged mutations [180,181].

The crystal structure of ETR bound RT revealed an important hydrogen bond interaction between K101 and the central ring of the inhibitor [182]. Additionally, the benzonitrile group is positioned in a pocket formed by V106, P225, F227, L234, P236, and Y318 while aromatic side chains of Y188, F227, and W229 residues position towards the dimethylcyanophenyl group of ETR [180,182]. These structural data have also revealed no change in the binding mode of ETR in the presence of K103N mutation and actually N103 side chain makes van der Waals interactions with the inhibitor. This observation is also validated by the drug sensitivity assays resulting in sustained activity against the frequently observed double drug-resistant mutations, K103N/Y181C and K101E/K103N [158]. The resistance profile of ETR has been explored and L100I, V179F/I, Y181C, G190E, M230L, and Y318F mutations were identified from the ETR-resistant virus isolates [179,183]. Furthermore, these experiments indicated that high-level resistance to ETR could only be accomplished by accumulating at least two mutations such as Y181C/V179F, L100I/K103N/Y181C, and Y181C/K103N/V179I. Clinical studies have also shown that combinations of three or more mutations including V90I, A98G, L100I, K101E/H/P, V106I, E138A/K/G/Q, V179D/F/T, Y181C/I/V, G190A/S, and M230L reduce the virological response to ETR [165,184,185,186,187]. Among these mutation positions E138 and V179 are novel and specific to the later-generation NNRTIs conferring low-level resistance to ETR [188,189]. Moreover, emergence of E138K after Y181C mutation increases the ETR susceptibility compared to Y181C mutant alone [190]. Interestingly, several connection subdomain mutations, E399D and N348I, have been identified with diminished ETR susceptibility [191,192,193].

The resistance mechanism against RPV resembles ETR since their chemical structures with conformational flexibility and adaptability are similar [182]. Therefore, RPV has a high resistance barrier like ETR as well. Under the selective pressure from RPV, several mutations have emerged including V90I, L100I, K101E/P, V106A/I, V108I, E138A/G/K/Q/R/V, V179L/F/I, Y181C/I/V, Y188L, V189I, G190E, H221Y, F227C, and M230I/L [189,194]. In the previous subsection, we have specified that Y181C and the Y188 mutations cause resistance for the first-generation NNRTIs due to loss of the aromatic ring stacking interactions; such interaction losses would not affect the binding of later-generation NNRTIs [195]. A recent *in vitro* study demonstrates that addition of E138 mutations on top of Y181C mutation shows no significant enhancement in resistance to RPV or ETR [196]. Also, E138K is determined to be the most predominant mutation besides K101E for both RPV and ETR [196,197]. K101E confers high resistance to RPV and compensates for viral replication deficiency and enzyme processivity associated with M184I [197]. Additionally, E138A/G/K/Q/R mutations alone have modest resistance profile against RPV, however when combined with M184I it significantly decreases susceptibility to RPV and ETR [189]. Earlier biochemical studies suggested that E138K substitution alters the RPV dissociation and association equilibrium, thus reduce RPV susceptibility [198]. The same study also showed that M184I has decreased catalytic efficiency because of a decreased binding affinity of dNTP to the mutant RT. According to the ECHO and THRIVE clinical trials, virological failure was seen most frequently in the presence of E138K and M184I double mutant upon RPV treatment combined with emtricitabine/tenofovir [199]. In a different note, it has been shown that N348I connection domain mutation alone or along with the M184V mutation could prevent or delay the emergence of E138K in patients under RPV selection pressure and decrease the replication capacity of E138K virus via reducing the catalytic efficiency and RNase H activity of RT [193].

### 4.3. Resistance to HIV-1 Protease Inhibitors

**Figure 6:**
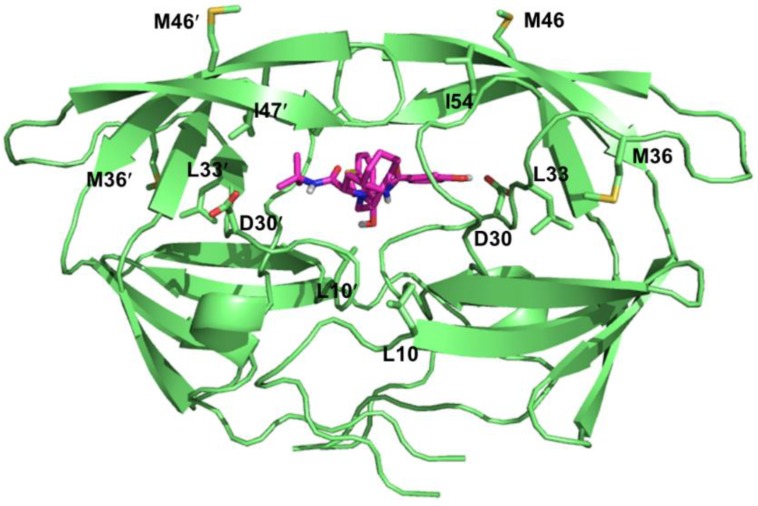
Detailed view of the protease inhibitor binding pocket. Nelfinavir (NFV) is shown as magenta sticks and the NFV-resistant mutation sites are shown in green sticks. PDB 1OHR [218].

HIV-1 protease is a viral aspartic protease that cleaves Gag and Gag-Pol polyproteins into individual functional proteins necessary for viral maturation [200]. Currently, there are nine FDA-approved protease inhibitors (PIs) for the treatment of HIV infection: Atazanavir (ATV), Darunavir (DRV), Fosamprenavir (FPV), Indinavir (IDV), Lopinavir (LPV), Nelfinavir (NFV), Saquinavir (SQV), Tipranavir (TPV), and Ritonavir (RTV). These inhibitors have been rationally designed with the aid of structure-based drug design and synthesized according to the substrate transition model [201]. All approved PIs are peptidomimetics excluding TPV and bind to the active site of the protease as competitive inhibitors (Figure 6) [202,203]. Additionally, RTV is only used as a boosting agent partner (only NFV could be used without the boosting agent RTV) due to its various side effects [204]. RTV is a potent inhibitor of cytochrome P450 3A4 (CYP3A4), thus increases the plasma concentrations of the partner protease inhibitors that are degraded *in vivo* by CYP3A4 [205].

PIs are essential in HAART regimens. However, development of drug resistance is an important hindrance for the success of protease inhibitor therapy [206]. HIV confers resistance by accumulating mutations within the enzyme that leads to reduced binding affinity of the inhibitors while maintaining the natural substrate binding interactions (Table 4). The HIV-1 protease is a homodimer consisting of 99 amino acid monomers [207]. Each monomer contributes to substrate binding, where the residues 25-32, 47-53, 76 and 80-84 form the substrate binding pocket. The majority of the drug-acquired mutations arise at the active site pocket as well as the surrounding residues since the PIs have similar binding modes compare to substrates. Common primary resistance mutations include D30N, V32I, L33F, M46I/L, I47A/V, G48V, I50L/V, V82A/F/T/S/L, I84V, and L90M [208,209]. While some of these mutations are selected by a specific PI such as the D30N substitution occurrence during the NFV therapy [210], I84V mutation causes cross-resistance to all PIs [211]. These primary resistance mutations could reduce the catalytic activity via changing the electrostatic and hydrophobic interactions networks to the inhibitor molecule from the mutated amino acids [208,212,213,214]. Moreover, these mutations also initiate several structural rearrangements throughout the protein globally to adjust the inhibitor binding [215,216]. All the resistance mutations are listed on Table 4 according to the latest update on the drug resistance mutations in HIV-1 [217].

Contrary to primary mutations that result in loss of viral fitness, additional secondary mutations at the neighboring non-active site pocket residues compensate for the impaired protease activity via increasing the activity and/or the stability of the protein [219,220,221]. Besides the secondary mutations, several other molecular mechanisms play an important role in the recovery of viral fitness as well. These mechanisms include the co-evolution of Gag and Gag-Pol cleavage sites [222,223,224,225,226]; mutations that alter the Gag-Pol frameshift leading to an increased expression of pol products [227]; Gag mutations at the non-cleavage sites improve the recovery of viral fitness by improving the access of the viral protease to the cleavage sites [228,229,230].

Second generation PIs (LPV, FPV, TPV and DRV) possess better pharmacological profiles, less severe side effects and demonstrate improved resistance profiles against the multidrug-resistant protease variants compare to first generation PIs [231,232,233,234]. Detailed knowledge of the protease structure has led to the design of new generation inhibitors towards the drug-resistant variants with enhanced binding affinity and a higher genetic barrier for further resistance mutations [235]. For example, a recent study indicated that HIV-1 protease isolates accumulated mutations in the range of 19 to 32 associated with decreased susceptibility to PIs [211]. Novel protease inhibitor designs illustrate promising antiviral activity profiles against the multidrug resistant HIV-1 variants [236].

**Table 4:** Major Protease Inhibitor Resistance Mutations.

Consensus	Amino acid mutations							
	ATV/r	DRV/r	FPV/r	IDV/r	LPV/r	NFV	SQV/r	TPV/r
L10	I, F, V,C		F,I,R,V	I,R,V	I,R,V	F,I	I,R,V	I
V11		I						
G16	E							
K20	R,M,I,T,V			M,R	M,R			
L24	I			I	I		I	
D30						**N**		
V32	I	I	**I**	I	I			I
L33	I,F,V	F	F		F	F		F
E34	Q							
M36	I,L,V			I		I		I,L,V
K43								T
M46	I,L		I,L	I,L	I,L	I,L		I,L
I47	V	V,A	**V,A**	V,A	V,A	V,A		V,A
G48	V,M				V,M	**V,M**	**V,M**	
I50	**L**	V	**V**		V			
F53	L,Y				L			
I54	L,V,M,T,A	M,L	**L**,V,**M**,T,A	V,T,A,L,M	V,L,A,M,T,S	V,T,A,L,M	V,L,T,A,M	A,M,V,T
Q58								E
D60	E							
I62	V						V	
L63					P			
I64	L,M,V							
H69								K,R
A71	V,I,T,L			V,T	V,T	V,T	V,T	
G73	C,S,T,A		S	S,A	S		S	
L74		P						P
L76		V	**V**	V	V			
V77				I		I	I	
V82	A,T,F,I,S	F	A,**F**,S,T	A,F,T,S	A,F,T,S	A,F,T,S	A,F,T,S	L,T,S
N83								D
I84	**V**	V	**V**	**V**	V	**V**	**V**	V
I85	V							
N88	**S**			S		**D,S**	S	
L89		V						I,M,V
L90	M		M	M	M	**M**	M	
I93	L,M							

The resistance mutations are compiled from the updated International Antiviral Society-USA (IAS-USA) and Stanford HIV drug databases. Mutations that are highlighted in bold represent the reduced susceptibility or virological response.

### 4.4. Resistance to HIV-1 Integrase Inhibitors

HIV-1 integrase is a 32 kDa protein that originates from the cleavage of the Gag-Pol polypeptide precursor mediated by the viral protease [237]. This viral protein consists of three different domains including N- and C-terminal domains and a catalytic core. All of the domains of integrase are required for its integration activity and this viral protein functions in a multimeric form [238]. HIV-1 integration process occurs in three consecutive steps: formation of the preintegration viral DNA complex, 3'-processing, and strand transfer [239]. The integrase enzyme catalyzes two major reactions after binding to the virally encoded DNA and joints it with host chromosomal DNA. The first reaction is 3'-processing, which consists of the endonucleolytic cleavage of the 3' ends of the viral genome. The cleavage occurs at the conserved CpA dinucleotide motif, thereby generating CpA-3'-hydroxyl DNA ends that are required for the next strand transfer step. The second catalysis step is strand transfer reaction involves the ligation of the viral 3'-hydroxyl DNA ends to the 5'-DNA phosphate of a host chromosome thus leading to the insertion of viral DNA into the host DNA [240,241]. Clinically approved integrase inhibitors, Raltegravir (RAL), Elvitegravir (EVG), and Dolutegravir (DTG), bind to the catalytic core (amino acids 50-212) of the integrase and target the integration process at the strand transfer step [242]. Catalytic core contains D64, D116, and E152 active site residues that coordinate the divalent cations required for 3'-processing, and strand transfer [243]. Drug resistant mutations against integrase inhibitors are determined via drug selection and viral fitness studies. Table 5 lists the major integrase inhibitor resistance mutations against the clinically approved drugs.

Resistance to RAL generally arises from three major mutation sites at positions Y143, Q148, and N155 with the accumulation of one or more additional residue changes in their vicinity [244]. Clinical data on RAL resistance has demonstrated the development of two main Q148 and N155 resistance pathways producing high-level phenotypic resistance to all approved integrase inhibitors [245,246]. These mutation pathways are thought to be mutually exclusive and do not appear in the same viral genome. Even though the Q148 and N155 pathways are predominant, there is another Y143 pathway that has been detected in patients [247]. Q148 pathway includes Q148H/K/R substitutions accompanied with subsequent mutations like L74M, E92Q, T97A, G136R, E138A/K, G140A/S, and V151I [244]. Alternatively, the N155 pathway predominantly consists of N155H mutation and occasionally associated with L74M, T97A, E157Q, and G163K/R [247]. The infrequent Y143 pathway consists of Y143C/H/R mutations and L74A/I, T97A, G163K/R accessory mutations [245,246]. Furthermore, *in vitro* assays showed that G140S/Q148H double mutant and E92Q mutation desensitized the RAL activity by 7-8 fold and the N155H mutant was 14 times more resistant to RAL compare to WT integrase [248]. It has been also suggested that G140S substitution in the double mutants increases the viral fitness produced by Q148K and Q148H [249,250].

EVG was approved in 2012 and formulated as a partner agent to FTC and EFV in Stribild^®^ (quad pill) together with cobicistat as a pharmacokinetic enhancer for EVG[251]. Unfortunately, EVG shows a similar resistance mutation profile in comparison with RAL, because the major mutations at Q148 and N155 pathways along with their accessory mutations have also been selected by EVG [252,253]. However, the third Y143 mutation pathway does not confer EVG cross-resistance. In contrast, there are specific mutation sites that confer resistance to only EVG such as T66 position mutations (T66I/A/K). In addition, E92Q mutation, which is located at the active site of integrase, causes higher resistance to EVG than RAL [254].

DTG is the latest approved drug against HIV infection and effective against the RAL and EVG resistant clinical isolates of HIV-1 including Q148K, N155H, Y143, and G140S/Q148H mutations [255]. Therefore, it has been suggested that DTG has a higher genetic barrier to resistance than RAL and EVG [252]. One reason for this outcome could be the determined slow dissociation rate (*k*_off_) of DTG from integrase-DNA complex in comparison to RAL and EVG [256]. Although DTG retains its antiviral activity against majority of the single mutation variants [257], R263K mutation is found to confer low-level resistance to DTG and cause decreased integration in cell culture [258]. The same mutation has been reported in a clinical study [259]. Furthermore, R263K mutation in combination with H51Y showed decreased susceptibility to DTG while declining viral fitness via reduced integration and impaired viral replication [260]. Secondary mutations M50I and E138K compensate for this reduced viral replication. Another resistance mutation that has been selected in cell culture after DTG treatment is G118R mutation [261]. Remarkably, viruses containing R263K and G118R DTG resistant mutations alone or along with secondary mutations were impaired in their ability to develop resistance against 3TC and NVP in *in vitro* studies [262]. In summary, recent insights on drug resistance have suggested that cross-resistance is a big hurdle and to avoid this problem more second generation integrase inhibitors like DTG should be designed.

**Table 5:** Major Integrase Inhibitor Resistance Mutations.

Consensus	Amino acid mutations		
	RAL	EVG	DTG
T66	A	**I**,**A**,**K**	
L74	M		
E92	**Q**	**Q**,G	Q
T97	A	A	
E138	A,K	K,A	A,K
G140	A,S,C	A,S,C	A,S,C
Y143	**R**,**H**,**C**		
S147		**G**	
Q148	**H**,**K**,**R**	**R**,**H**,**K**	H,R,K
N155	**H**	**H**	

The resistance mutations are compiled from the updated International Antiviral Society-USA (IAS-USA) and Stanford HIV drug databases. Mutations that are highlighted in bold represent the reduced susceptibility or virological response.

### 4.5. Resistance to Viral Entry Inhibitors

HIV entry is a combination of multi-step interrelated processes that involves coordinated multiple sequential interactions between the viral gp120 and gp41 and host surface proteins [263]. Even though there are several potential targets during the entry processes, only two different classes of entry inhibitors were approved by FDA to block viral entry into cells. The first drug Maraviroc (MVC) is a chemokine receptor antagonist targeting the interaction between gp120 and CCR5 chemokine coreceptor after gp120 binds to the CD4 receptor on the host cell surface [264]. Two classes of viruses were identified and classified as T-tropic and M-tropic viruses infecting CD4+ T cells and macrophages, respectively [265,266,267]. M-tropic viruses use CCR5 as the preferred coreceptor [268], whereas T-tropic viruses utilize CXCR4 as a coreceptor for viral entry [269]. MVC is a selective CCR5 antagonist that targets host protein contrary to the other anti-HIV drugs [270]. MVC binds to an allosteric hydrophobic pocket formed by the transmembrane helices of CCR5 and inhibits HIV-1 entry through altering the conformation of the receptor so that it can no longer be recognized by gp120 [271,272]. Despite its successful antiviral efficacy in clinical trials [273,274], several *in vitro* and *in vivo* studies demonstrated diverse resistance mechanisms against MVC therapy. One resistance mechanism that HIV utilizes to evade MVC treatment is the tropism switch by utilizing CXCR4 coreceptor instead of CCR5 to enter the cell [275]. According to the *in vitro* studies, selection of CXCR4 tropic viruses may not be main mechanism of escape [276,277,278]. However, the major resistance mechanism against MVC is emergence of multiple mutations in the HIV-1 envelope protein that allow the virus to use the MVC occupied CCR5 coreceptor [279,280]. The majority of these mutations were mostly located in the V3 loop region of gp120 [279,281,282]. In conclusion, the results from these studies suggest that MVC-resistant virus could bind to CCR5 coreceptor both in a drug free conformation and MVC-bound conformation. Moreover, HIV-1 may switch coreceptor usage to CXCR4 if CXCR4 using species were present during the initial drug treatment period [275]. 

The other class of entry inhibitor, Enfuvirtide, is a fusion inhibitor that targets the fusion activity of gp41 [283]. HIV-1 gp41 is responsible for anchoring the envelope complex to the cell membrane, thereby bringing the virus and target cell into close proximity and assisting their fusion by further conformational rearrangement in its structure. During membrane fusion, heptad repeat 2 (HR2) region of gp41 folds onto the heptad repeat 1 (HR1) hydrophobic region thereby shortening the molecule. Enfuvirtide is a 36 amino acids long synthetic peptide derived from the C-terminal region of HR2 [284]. Enfuvirtide shows its antiviral activity through binding to HR1 region of gp41 and blocking the virus from infecting the cell. Predictably, viral resistance to Enfuvirtide emerges through a stretch of amino acids that are located in the HR1 region as shown in Table 6 [285].

**Table 6:** Major Mutations in The Envelope Gene Associated with Resistance to Enfuvirtide.

Consensus	Amino acid mutations
	Enfuvirtide
G36	D,S
I37	V
V38	A,M,E
Q39	R
Q40	H
N42	T
N43	D

Specifically, amino acid positions G36, I37, and V38 were shown to confer resistance or sensitivity to Enfuvirtide. In vivo and other *in vitro* studies have confirmed the mutations within these gp41 residues 36-38, as well as some other mutation sites along the HR1 region [286,287,288,289,290,291,292,293]. These mutations emerge quickly upon drug treatment and are mostly found in various HIV subtypes. In addition, several combinations of two mutations were detected that lead to strong resistance to the inhibitor such as G36D/N42T, G36V/N42D, I37M/N43D, V38A/N42D, V38A/N42T, V38A/L44D, V38E/N42S, and Q41R/N43D [294,295,296,297]. The resistance mutations on HR1 region that Enfurvitide binds destabilize the six-helix bundle structure of gp41 and thus causing decreased binding affinity for the inhibitor [285,296,298]. While efficient discrimination against Enfurvitide could be established by these mutations, they could also reduce the viral fitness by disrupting the interactions between the mutated HR1 and the endogenous HR2 [289,299,300]. In order to address these destabilizing HR1 mutations, new mutations in HR2 region could emerge to compensate for the fitness loss later in the treatment. Several x-ray crystallographic studies have shown that HR2 mutations could enhance the stability of the six-helix bundle via direct interactions to the HR1 mutated residue [301,302].

## 5. Monitoring Antiretroviral Drug Resistance

HIV treatment options have become more effective at delaying the drug resistance evolution during the last decades. However, there is still room to improve the emergence of resistance since acquired drug resistance still occurs in the clinic. Routine viral load monitoring and viral genotyping help the clinicians to find better drug choices and combinations with minimum risk of drug resistance. It also prevents unnecessary treatment switches and accumulation of drug resistance in patients. Especially in developing countries, low-cost technologies to diagnose and monitor HIV infection are crucial in health care settings [303]. For example, there has been a slight increase in the prevalence of NNRTI resistance mutations, which was attributed to infection with resistant viruses in low to middle income countries where NNRTI regimens are very common and resistance monitoring is absent [304]. Therefore, more efforts have been directed to develop point-of-care (POC) technologies that are affordable, robust, portable and easy to use with high accuracy results to aid the decision making for patients who are failing therapy in resource-limited settings. For instance, a new nucleic acid-based POC platform, simple amplification-based assay (SAMBA), has been described for HIV-1 testing [305]. The SAMBA test relies on isothermal amplification with a visual readout, which makes it suitable for resource poor settings. All the currently available POC technologies have been outlined in a recent review article [303]. Viral load monitoring is important to identify individuals who are not adherent to their treatment and to switch treatment options according to the changes in viral load over time [306]. If the viral load rises above 200 copies/mL, it might be sign of emergence of drug resistance mutations. For HIV-1 genotyping, there are several commercial or laboratory-developed tests are available to detect the resistance associated mutations. These tests mainly utilize PCR amplification and nucleotide sequencing of viral genes such as reverse transcriptase, protease, and integrase [307]. The WHO and International AIDS Society-USA Panel recommends antiretroviral drug testing as a routine management of the viral infection in developing countries. Coreceptor tropism testing is also becoming available since new drugs are entering to the pipeline. More effort should be placed in improving the sensitivity and accuracy of these assays to better detect the minor variants that might be clinically significant.

## 6. Conclusions and Future Directions

Recent clinical data and experimental studies have demonstrated that HIV-1 infection could be successfully treated with the minimized resistance mutations [308,309]. These new drugs have superior antiviral efficacy with high viral suppression and higher genetic barriers against the evolution of drug resistance. Moreover, the improved pharmacokinetics and bioavailability properties of currently available drugs have allowed simplification of their dosing regimens in combination therapies. Even though there is a widespread shift in basic research towards finding a cure for HIV or developing new drugs or formulations for prevention, we still have to treat infected patients with limited therapy options because of the developed drug resistance. Viral load monitoring is still not available for everyone in limited resource settings. These patients could stay on the failing treatment and acquire more drug resistance mutations, which could compromise the second-line treatment options as well. In order to solve the drug resistance problem, the availability of newer drugs and monitoring needs to be improved drastically. Also, there is always a possibility of therapy failure for current drugs and no one can predict if these drugs will retain their efficacies for many years in the future. Therefore, we still need to put a lot of effort on finding new drugs with novel mechanism of actions and exploit novel druggable viral targets that would give us the upper hand in the battle against HIV infection.
